# Confidence interval methods for antimicrobial resistance surveillance data

**DOI:** 10.1186/s13756-021-00960-5

**Published:** 2021-06-09

**Authors:** Erta Kalanxhi, Gilbert Osena, Geetanjali Kapoor, Eili Klein

**Affiliations:** 1 Center for Disease Dynamics, Economics and Policy (CDDEP), Washington, DC USA; 2 Johns Hopkins University, Baltimore, MD USA

**Keywords:** Antimicrobial resistance, Confidence intervals, Data correlation, Cluster-robust errors

## Abstract

**Background:**

Antimicrobial resistance (AMR) is one of the greatest global health challenges today, but burden assessment is hindered by uncertainty of AMR prevalence estimates. Geographical representation of AMR estimates typically pools data collected from several laboratories; however, these aggregations may introduce bias by not accounting for the heterogeneity of the population that each laboratory represents.

**Methods:**

We used AMR data from up to 381 laboratories in the United States from The Surveillance Network to evaluate methods for estimating uncertainty of AMR prevalence estimates. We constructed confidence intervals for the proportion of resistant isolates using (1) methods that account for the clustered structure of the data, and (2) standard methods that assume data independence. Using samples of the full dataset with increasing facility coverage levels, we examined how likely the estimated confidence intervals were to include the population mean.

**Results:**

Methods constructing 95% confidence intervals while accounting for possible within-cluster correlations (Survey and standard methods adjusted to employ cluster-robust errors), were more likely to include the sample mean than standard methods (Logit, Wilson score and Jeffreys interval) operating under the assumption of independence. While increased geographical coverage improved the probability of encompassing the mean for all methods, large samples still did not compensate for the bias introduced from the violation of the data independence assumption.

**Conclusion:**

General methods for estimating the confidence intervals of AMR rates that assume data are independent, are likely to produce biased results. When feasible, the clustered structure of the data and any possible intra-cluster variation should be accounted for when calculating confidence intervals around AMR estimates, in order to better capture the uncertainty of prevalence estimates.

**Supplementary Information:**

The online version contains supplementary material available at 10.1186/s13756-021-00960-5.

## Background

Antimicrobial resistance (AMR) represents one of the greatest global health challenges today, resulting in over two million antimicrobial-resistant infections and an estimated 35,000–162,000 deaths annually in the United States [1–4]. The World Health Organization’s (WHO) latest report, based on AMR data from 66 different countries, paints an alarming picture on the status of AMR across the world, with an increasing number of countries reporting high rates of resistance among antimicrobials used to treat common infections [5, 6]. Increasing access and use of antimicrobials in many low- and middle-income countries, as well as wide-scale improper use in higher-income countries, is driving the growing resistance to antimicrobials around the world [7–9]. The problem of resistance is compounded by the slow introduction of new antimicrobials [10, 11]. However, despite the growing burden of resistance and the fact that estimates for many geographical regions are based on limited numbers of samples, there has been a lack of attention paid to estimating the uncertainty of these estimates.

Assessment of the burden of resistance in a country (or region) is typically derived from analysis of routine antimicrobial susceptibility testing (AST). However, since not every AST result is available, statistical methods are used to estimate resistance rates for the selected region along with 95% confidence intervals (CI) denoting the uncertainty in the estimate. Besides sampling issues, for many countries susceptibility data comes from disparate sources with respect to quality, testing methods, and socio-demographic status, all of which can bias inter-laboratory comparisons.

The majority of studies construct CIs for AST data based on a binomial probability function which assumes data independence (i.e. all isolates have the same probability of testing positive and the results of each analysis are unrelated) [7, 12–14]. However, this is likely to introduce bias in the measure of uncertainty as isolates are unlikely to be independent because (1) each facility draws from different patient populations, (2) each laboratory follows different processes and uses different equipment, and (3) the criteria for ordering AST differs for each facility. Failure to account for these differences and control for within-cluster correlations can lead to underestimation of standard errors and misleadingly narrow confidence intervals [15]. Here, we evaluate the efficacy of the binomial probability function to define the level of uncertainty in a sample of antibiotic resistance data. We assess the likelihood that CIs generated by the binomial probability function (Wilson score and Jeffreys interval) and a transformed method (Logit), which operate under assumption of data independence, contain the true population mean. Then we evaluate the performance of additional methods such as Survey and adjusted versions of aforementioned methods that take into account the variation between data sources and the clustered structure of the data in CI construction [16–18].

## Methods

### Antibiotic resistance data

We used 2011 data from The Surveillance Network (TSN) Database USA (Eurofins Medinet, Chantilly, VA, USA), a repository of AST results which has been previously used to evaluate antimicrobial resistance patterns [19]. The AST data in the TSN database were derived from routine diagnostic testing using standards established by the Clinical and Laboratory Standards Institute (CLSI), and approved by the US Food and Drug Administration [20]. In this study, we evaluated the construction of confidence intervals for proportions of *Staphylococcus aureus* isolates that were non-susceptible to one of three antibiotics: Oxacillin, Rifampin and Penicillin. These three were chosen to assess antibiotic-pathogen combinations that included low (< 1%), high (~ 50%) and very high (~ 90%) resistance rates. Additionally, we evaluated a set of WHO priority pathogen/drug combinations: *Klebsiella pneumoniae* and Ceftriaxone, *Pseudomonas aeruginosa* and Imipenem, and *Acinetobacter baumannii* and Imipenem [21]. As not every facility submitted data for every pathogen/drug combination, the complete dataset included isolates from up to 381 facilities geographically spread across up to 33 states in the United States. Isolates that were resistant (R) or of intermediate susceptibility (I) were considered non-susceptible isolates.

### Data analysis

To assess how facility sample selection can affect the uncertainty of the estimated non-susceptibility rate, we computed the rates of non-susceptibility by repeatedly sampling from the total number of facilities in the TSN dataset. By randomly selecting different numbers of facilities (10, 25, 50 and 100), we attempted to approximate the scenario where AMR prevalence in a country is based on a sample of laboratories. We performed 500 iterations where we sampled without replacement for each sample size and estimated the proportion non-susceptible ($\widehat{{\text{p}}}$) and then constructed CIs for seven different methods (Table 1). The different methods can be divided into two different categories: (1) methods which assume isolate independence and (2) methods that assume that isolates are likely to be correlated at the facility level. A description of each model follows:

**Table 1:** Methods for estimating confidence intervals

Methods	Description	Error estimations
Wilson score interval	Binomial proportion CI	Standard
Jeffreys interval	Binomial proportion CI	Standard
Logit	Transformed method	Standard
Wilson Robust	Adjusted method	Cluster-robust
Jeffreys Robust	Adjusted method	Cluster-robust
Logit Robust	Adjusted method	Cluster-robust
Survey	Survey design method	Standard

#### Wilson score interval and Jeffreys interval

The Wilson score and Jeffreys intervals, are two methods that estimate the probability of an event occurring in a population, provided the events’ outcome follows a binomial distribution [13]. The Wilson score interval is close to the nominal level of 95% for a 95% confidence interval, hence its wide use in many AMR studies [7, 22, 23]. Jeffreys and Wilson score intervals are considered to have similar performances and are recommended for use in estimations involving small sample sizes (n < 40) [24]. Both methods rely on the assumption of data independence and estimate variability through the calculation of standard errors. We compare the CI from these methods under assumptions of independence with versions that take account of potential differences by facility by including cluster-robust errors and inter-cluster variation [25]. We call these modified methods as Wilson Robust and Jeffreys Robust.

#### Logit

Logit is a transformation method in which proportions are log-transformed to stabilize variance for the construction of confidence intervals [16]. Though not commonly used in AMR studies, the transformations could normalize distribution and stabilize variance for samples derived from populations that may not have the same variance or standard deviation [26], which is common. We include a version that does not account for inter-cluster variation when samples come from different sources and a modified version where cluster-robust errors account for inter-cluster variation, which we call Logit Robust.

#### Survey

An alternative means of assessing the resistance rate in a population is to consider the data like a survey. In particular, two feature characteristics of survey design, clustering and stratification, allow the data to more accurately account for potential clustering at the facility level [27]. Clusters represent the Primary Sampling Unit (PSU) from where the data are collected from (i.e. different laboratories), and this stratification enables accounting for intra-cluster correlation during data analysis. Furthermore, additional stratification enables the grouping of clusters according to shared qualities (i.e. quality or geographical spread). In the context of this study, the CI were constructed using the *svy* command; the facilities were considered as PSU and the analyses were stratified according to the geographical spread represented by the US state in which each facility is located.

### Coverage probability

CIs represent estimates, which should ideally contain the true population estimate at the stated level (i.e. 95% of the time). As they are constructed based on data from a sample of the total population, they are susceptible to sampling variation and may, at times, not include the true mean value; however, the assumption is that for a 95% CI there is a 95% level of certainty that it includes the true population mean. Furthermore, with repeated sampling, there is a 95% probability that the CI for that sample should cover the true mean. To evaluate the performance of each method, we estimated their coverage probability, a method which assesses the proportion of times that the true population estimate is included in the CI range when randomly sampling from a certain population. Coverage probability was calculated for each method as the proportion of all CIs of the samples that encompassed the overall non-susceptibility rate from all the reporting facilities.

Simulations for the calculation of coverage probabilities for each method were performed using Stata version 16.0 (StataCorp LP, College Station, TX, USA). Group and pairwise comparisons of coverage probabilities for the different methods were performed with non-parametric tests Kruskal–Wallis and Mann–Whitney–Wilcoxon test, respectively using R version 3.6.3. Results were considered statistically significant when the *P* value was less than 0.05.

## Results

The AMR data consisted of isolates collected in 2011 from 174 to 381 health facilities across 33 states in the US. The number of isolates tested from each facility spanned from one to thousands (Table 2) and the overall distribution of the samples across the states where the facilities were located is illustrated in Additional file 1: Fig. S1. The percentage of non-susceptible isolates for *S. aureus* (sum of isolates interpreted as resistant and intermediate) from across all the facilities was 1.2%, 48.3% and 92.3% for Rifampin, Oxacillin, and Penicillin, respectively (Table 2). Carbapenem resistance was 24.8% for *A. baumannii* isolates and 18.8% for *P. aeruginosa* isolates*,* while cephalosporin resistance was 7.0% *for K. pneumoniae* (Table 2).

**Table 2:** Susceptibility of pathogens to antimicrobial agents and sample distribution

Pathogen/antimicrobial agent	No. of isolates (%)			Distribution of isolates	
	Susceptible	Resistant	Total	No. of testing facilities (range of tested isolates)	No. of states
*S. aureus*/Rifampin	124,457 (98.8)	1466 (1.2)	125,923	168 (1–4977)	31
*S. aureus*/Oxacillin	71,788 (51.8)	71,605 (48.3)	148,393	174 (1–5183)	33
*S. aureus*/Penicillin	600 (7.7)	102,980 (92.3)	111,580	156 (1–4979)	33
*K. pneumoniae*/Ceftriaxone	37,227 (93%)	2802 (7.0)	40,029	358 (1–4162)	31
*A. baumannii*/Imipenem	2651 (75.2)	873 (24.8)	3524	227 (1–1299)	29
*P. aeruginosa*/Imipenem	35,934 (81.2)	8319 (18.8)	44,253	381 (1–3604)	31

**Fig. 1:**
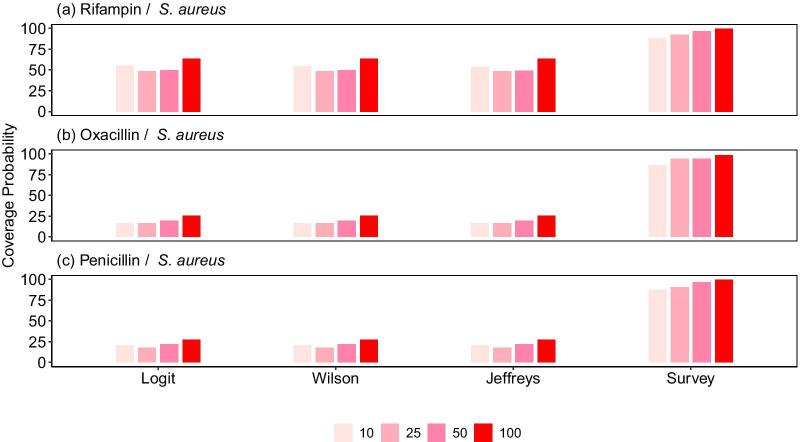
The effect of proportion value and number of sampling facilities on coverage probabilities. Coverage probabilities for all methods were calculated for estimation of confidence intervals for different proportions of resistant isolates, as indicated by the varying degrees of resistance to **a** Rifampin (1.2%), **b** Oxacillin (48.3%) and **c** Penicillin (92.3%), and for samples collected from an increasing number of facilities (10, 25, 50 and 100)

When constructing CIs from data collected from 10 facilities, the coverage probabilities for the Wilson score method were 55% for *S. aureus*/Rifampin and only 16% and 21% for *S. aureus*/Oxacillin and *S. aureus*/Penicillin, respectively (Fig. 1, Additional file 1: Table S1). Increasing the sample size (by sampling from an increasing number of facilities), to 25 or 50 was not associated with an increase in coverage for the standard methods (*P* = 0.18 and *P* = 0.32), though there was a modest increase in coverage probability when the number of facilities sampled was increased to 100 (*P* < 0.01). However, as illustrated by the case of Rifampin-resistant isolates, the coverage probability of these methods was significantly greater for proportions nearing zero (*P* < 0.01). Compared to the Wilson Score, Jeffreys, and Logit, the Survey method was significantly more likely to contain the population mean (*P* < 0.01) (Fig. 1).

**Fig. 2:**
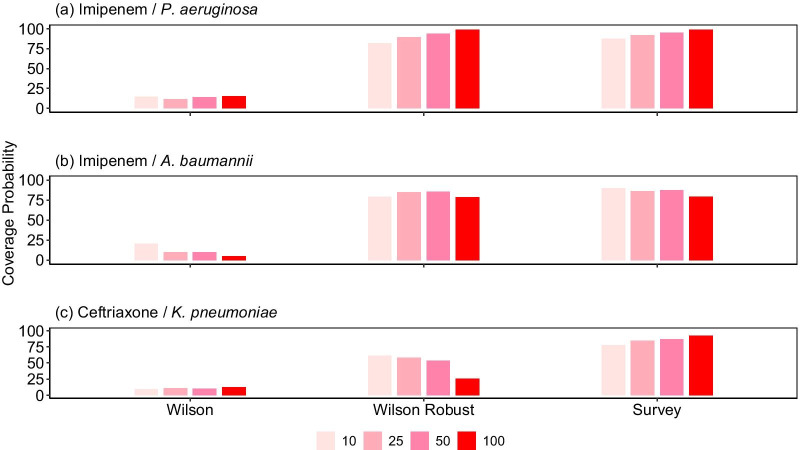
Effect of cluster-robust errors on coverage probabilities. The coverage probabilities of the standard and adjusted Wilson methods (using cluster-robust errors) and the survey method were calculated for estimation of confidence intervals for AMR estimates for various pathogen and drug combinations: **a** *P. aeruginosa* and Imipenem, **b** *A. baumannii* and Imipenem, **c** *K. pneumoniae* and Ceftriaxone, using data collected from an increasing number of facilities (10, 25, 50 and 100)

Accounting for the potential inter-facility clustering for the Wilson Score, Jeffreys, and Logit methods resulted in significantly greater coverage probabilities (*P* < 0.01) (Additional file 1: Fig. S2). Results were similar for other pathogen and drug combinations; the use of robust errors increased probability coverage when constructing CIs for carbapenem-resistant (*A. baumannii* and *P. aeruginosa*) and 3rd generation cephalosphorin-resistant (*K. pneumoniae*) pathogens (Fig. 2).

**Fig. 3:**
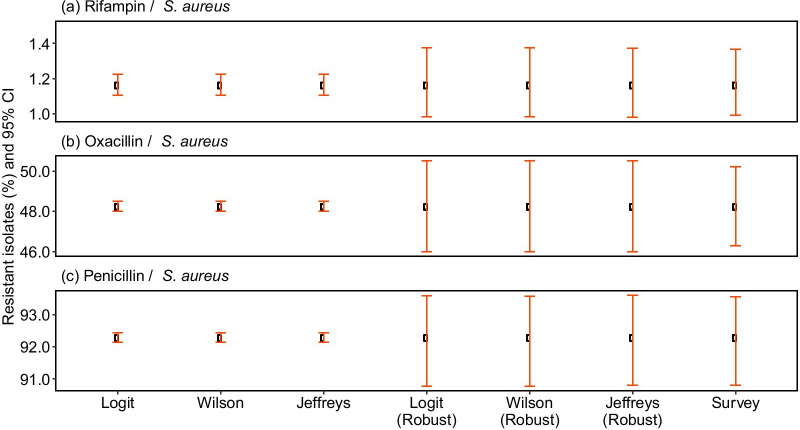
Effect of accounting for intra-cluster correlation on confidence interval widths. Proportions of **a** Rifampin-, **b** Oxacillin- and **c** Penicillin-resistant isolates from the entire dataset and respective 95% CI were estimated using standard methods (Logit, Wilson, Jeffreys), their modified versions employing cluster-robust errors, and Survey

**Fig. 4:**
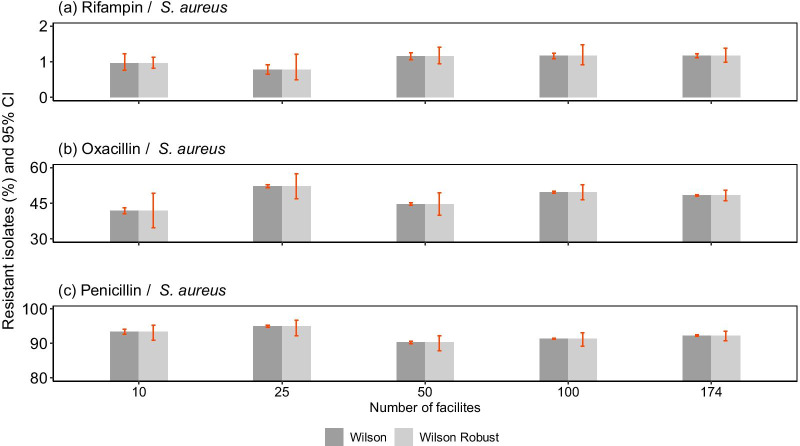
Effect of sample size on confidence interval widths. Proportions of **a** Rifampin- **b** Oxacillin- and **c** Penicillin-resistant isolates from increasing number of facilities, and their respective 95% CI were estimated using the Wilson score method and its adjusted robust version, employing cluster-robust errors

The robust version of the standard methods and the Survey method generated wider CIs (Fig. 3, Additional file 1: Table S2, Fig. S3). Applying the different methods to the overall population we found that the CIs for the robust methods were about 11 and 7 times larger than those for the standard methods for *S. aureus* with Oxacillin and Penicillin respectively, and about 4 times larger for Rifampin. For the Wilson Score method, the range increased from 0.1 to 0.4% for Rifampin, from 0.5 to 5.5% for Oxacillin, and 0.4% to 2.8% for Penicillin. The results were similar for the other methods. Additionally, the uncertainty displayed by CI widths, was found to be greater at smaller sample sizes and for proportions nearing zero (Fig. 4, Additional file 1: Figs. S4 and S5).

## Discussion

Assessing the burden of AMR across the world and especially in low- and middle-income countries is challenged by gaps in prevalence and geographical distribution, mainly due to lack of surveillance infrastructure and technical expertise [22, 28–30]. In countries where data are available, AMR outcomes are typically pooled to achieve national or regional averages [6], however, given the great variation in quality and geographical spread between health facilities and laboratories, this average may not be representative of population estimates [31]. Inference on population parameters based on data from a population sample are generally accompanied by 95% CIs as a measure of uncertainty. For AMR data, most studies assume the data follows a binomial distribution, and each result is independent from all other results. However, in reality outcomes from one health facility may be more comparable to each other than to those from other centers, implying a degree of data correlation. Thus, the violation of the data independence assumption often leads to confidence interval estimates that are too narrow and unlikely to contain the true proportion of resistance in the population.

By randomly sampling facilities included in a large dataset of AMR data from the US, we demonstrate the bias in uncertainty measurements that is likely a characteristic of most AMR data that are collected from multiple facilities. At low resistance levels, the bias in these results lessened, but was not mitigated. However, at high rates, the impact was quite drastic—the coverage probability was only around 25% in the non-clustered methods compared with over 80% even with the fewest number of facilities and closer to 95% as the sample numbers increased in the clustered methods. When accounting for clustering, the confidence intervals were 7–11 times larger on average compared to the non-clustered methods. Moreover, the difference in confidence interval widths between non- and cluster methods increased as the number of sampled facilities decreased from 174 to 10. For instance, using the Wilson method and sampling from 174 facilities, representing the entire dataset for *A. aureus*, the average spread of the confidence intervals for Oxacillin-resistant isolates increased from 0.5 in the non-cluster methods to 5% points in the clustered methods; however, the average confidence interval spread increased from 2.6 to 14 percentage points, when sampling from 10 facilities. Widening of the confidence intervals widths as the number of the sampling facilities is reduced, illustrates the increasing uncertainty when sampling from few units with great heterogeneity in the proportion of resistant isolates. In such instances, reporting of AMR estimates from each cluster, may be more appropriate than aggregating the samples.

While increasing the number of facilities, and thus the number of isolates in the sample, reduced the bias (i.e., increased the coverage probability in most cases), this difference was only marginal and not sufficient to remove the bias introduced by the violation of the data independence assumption. In some instances, as in the case of *K. pneumonia*, the increase in the number of sampling facilities led to a reduction of coverage probability. This is likely due to high geographical heterogeneity as the survey method, which accounts for this type of sampling, did not show this same pattern. However, confidence intervals for small proportions of resistant isolates (equal or less than 0.01), were wider and were associated with better coverage probabilities, even when data correlation was not taken into consideration. This observation was in line with findings from a previous study demonstrating (through simulations with real pharmacological data), that the Wilson score with continuity correction was recommended as one of the methods for constructing confidence intervals for very small proportions ranging from 0.001 to 0.1 [32]. Overall though, AMR prevalence estimates derived from aggregated data should include stratification of samples according to their source or other shared qualities, whenever possible. When this information is not available, alternative methods should be evaluated to improve the estimates. For example, a bootstrap analysis that resamples results with random clustering. Future studies should evaluate methods to account for this uncertainty bias when the number of labs is less than ten.

The fact that employing cluster-robust errors instead of standard errors led to a significant improvement in coverage and widening of the confidence intervals, suggests that facility-level differences matter. There are several potential reasons for these differences. The first is that each represents geographical differences in resistance. There is some evidence that local patterns of resistance may be important in *S*. *aureus* [33]. The second is that there may be differences in practice patterns that determine patient culture probability. Variation in practice patterns are well documented in medicine [34–36]. For example, one study of blood culturing practices found wide variation in the rate of blood cultures per 1,000 patient days [37]. These variations in culturing practices could lead to large differences in resistance rate estimates. Finally, there may be differences in the quality of the culture or the laboratory. Contamination of samples with other organisms can affect resistance rates either by including estimates of organisms that are not clinically important or resulting in samples being rejected. In addition, the sensitivity and quality of laboratory instruments can vary widely. While this is likely less of a problem in high-income countries, there still remain differences in how samples are processed that could introduce biased differences. In many low-income countries however, resource constraints can result in drastic differences in laboratory quality.

The goal of this study was to assess the implications of the choice of methodology in CI construction. However, an important limitation of the robust error approach is that while it adjusts standard errors for correlated data, it has no impact on the AMR estimates themselves which are sensitive to data heterogeneity across the sampling facilities. This is especially important when there are significant differences in the number of samples processed in each facility, a scenario that would warrant the use of weighted analysis methods.

While we attempted to use representative and generalizable data to assess these methodologies, the dataset used in our simulations contains data solely from the US, where heterogeneity in data sources may be considerably lower than in the low- and middle-income countries. We used the TSN database though as it is one of the largest datasets available with high, representative coverage, which allowed us to simulate scenarios in which different numbers of facilities with different sample volumes and characteristics were chosen in the sampling frame. The results are important for estimating the burden of resistance in other settings, as they illustrate that even in settings with large geographic representation and high quality labs, large uncertainties remain in AMR estimates. Finally, we assumed that the entire dataset contained the “true” population mean—and estimated coverage probabilities were based on the mean value. While the dataset is large, it is itself a sample of the population. Biases in the dataset could constrain the implications of the results, but the larger point of the analysis is not affected by this limitation.

## Conclusions

The construction of confidence intervals is necessary for understanding the level of uncertainty in estimates of AMR prevalence. Methods that assume independence between samples are likely to be biased and underestimate the variance in the estimate. Therefore, to increase the likelihood that CIs contain the true population mean, AMR prevalence estimates derived from data aggregated across facilities should include stratification of samples according to their source, or other shared qualities whenever possible. In reality, information on data sources is not always available and it is not always possible to incorporate stratification of samples in the analysis. Hence, for this reason, it is important to specify the types of methods used in the construction of the confidence intervals and to recognize that they may not always include the true population resistant estimates.

## Supplementary Information

**Additional file 1**. AMR estimates and confidence intervals for various pathogen-drug combinations.

## Data Availability

The datasets analyzed during the current study were made available to CDDEP through an exclusive licensing agreement with The Surveillance Network (TSN) and are not available for sharing due to the confidential nature of patient data.
