# Supplementary material for: Confidence interval methods for antimicrobial resistance surveillance data
Source: Antimicrob Resist Infect Control. 2021 Jun 9;10:91. doi: 10.1186/s13756-021-00960-5 (PMC8191092; doi:10.1186/s13756-021-00960-5)
Supplement: Supplementary file 1 — Additional file 1. AMR estimates and confidence intervals for various pathogen-drug combinations. [file 13756_2021_960_MOESM1_ESM.docx]

**Additional File 1**

**Confidence interval methods for antimicrobial resistance surveillance data**

Erta Kalanxhi^1^, Gilbert Osena^1^, Geetanjali Kapoor^1^, Eili Klein^1,2^

**Figure S1.** Distribution of tested isolates across the different states. (For *S. aureus*, isolates tested for resistance to penicillin were chosen as representative samples).

**Figure S2**. Effect of cluster-robust errors on coverage probabilities. The coverage probabilities of the adjusted methods (using cluster-robust instead of standard errors) were calculated for estimation of confidence intervals for AMR estimates using data collected from an increasing number of facilities (10, 25, 50 and 100).

**Figure S3. Effect of accounting for intra-cluster correlation on confidence interval widths**. Proportions of a) Rifampin-, b) Oxacillin- and c) Penicillin-resistant isolates from the entire dataset and respective 95% CI were estimated using standard methods (Logit, Wilson, Jeffreys), their modified versions employing cluster-robust errors, and Survey.

**Figure S4**. **Effect o**f **sample size on confidence interval widths**. Proportions of a) Rifampin- b) Oxacillin- and c) Penicillin-resistant isolates from increasing number of facilities, and their respective 95% CI were estimated using the Wilson score method and its adjusted robust version, employing cluster-robust errors.

**Figure S5**. **Sample size and confidence interval widths**. Proportions of a) Rifampin- b) Oxacillin- and c) Penicillin-resistant isolates from 3 and 6 facilities, and their respective 95% CI were estimated using the Wilson score method and its adjusted robust version, employing cluster-robust errors.

**Table S1**. Coverage probabilities for methods with standard and cluster robust errors estimating proportion of resistant *S. aureus* isolates in the TNS dataset

|  |  | **Antimicrobial agent** | | | | | | | | | | | | | |
| --- | --- | --- | --- | --- | --- | --- | --- | --- | --- | --- | --- | --- | --- | --- | --- |
|  |  | **Rifampin** | | | |  | **Oxacillin** | | | |  | **Penicillin** | | | |
| **No. of sampling facilities** |  | 10 | 25 | 50 | 100 |  | 10 | 25 | 50 | 100 |  | 10 | 25 | 50 | 100 |
| **Logit** |  | 55 | 48 | 50 | 64 |  | 16 | 17 | 19 | 26 |  | 21 | 18 | 22 | 27 |
| **Wilson** |  | 52 | 49 | 50 | 64 |  | 18 | 21 | 20 | 28 |  | 18 | 15 | 18 | 30 |
| **Jeffreys** |  | 51 | 50 | 50 | 64 |  | 18 | 21 | 20 | 28 |  | 17 | 15 | 18 | 30 |
| **Logit (Robust)** |  | 87 | 93 | 96 | 100 |  | 89 | 92 | 96 | 100 |  | 85 | 90 | 96 | 99 |
| **Wilson (Robust)** |  | 86 | 93 | 97 | 99 |  | 89 | 92 | 96 | 100 |  | 85 | 90 | 96 | 99 |
| **Jeffreys (Robust)** |  | 86 | 93 | 97 | 99 |  | 85 | 96 | 97 | 100 |  | 87 | 91 | 96 | 100 |
| **Survey** |  | 88 | 93 | 96 | 100 |  | 87 | 94 | 94 | 98 |  | 88 | 91 | 96 | 100 |

**Table S2**. Resistant isolates (%) and 95% CI for *S. aureus* estimated by different methods.

| **Antimicrobial agent** | **Rifampin** | **Oxacillin** | **Penicillin** |
| --- | --- | --- | --- |
| **Resistant isolates (%)** | 1.16 | 48.3 | 92.9 |
|  | 95% CI | 95% CI | 95% CI |
| ***Methods*** |  |  |  |
| **Logit** | 1.1-1.2 | 48.0-48.5 | 92.1-92.5 |
| **Wilson** | 1.1-1.2 | 48.0-48.5 | 92.1-92.5 |
| **Jeffreys** | 1.1-1.2 | 48.0-48.5 | 92.1-92.5 |
| **Survey** | 1.0-1.4 | 46.3-50.2 | 90.8-93.6 |
| **Logit Robust** | 1.0-1.4 | 46-50.5 | 90.8-93.6 |
| **Wilson Robust** | 1.0-1.4 | 46-50.5 | 90.8-93.6 |
| **Jeffreys Robust** | 1.0-1.4 | 46-50.5 | 90.8-93.6 |
